# Vitamin D Deficiency Is Not Associated With Growth or the Incidence of Common Morbidities Among Tanzanian Infants

**DOI:** 10.1097/MPG.0000000000001658

**Published:** 2017-09-22

**Authors:** Christopher R. Sudfeld, Karim P. Manji, Emily R. Smith, Said Aboud, Rodrick Kisenge, Wafaie W. Fawzi, Christopher P. Duggan

**Affiliations:** ∗ Department of Global Health and Population; † Department of Nutrition; ‡ Department of Epidemiology, Harvard T.H. Chan School of Public Health, Boston, MA; § Department of Paediatrics and Child Health; || Department of Microbiology and Immunology, Muhimbili University of Health and Allied Sciences, Dar es Salaam, Tanzania; ¶ Department of Division of Gastroenterology, Hepatology and Nutrition, Boston Children's Hospital and Harvard Medical School, Boston, MA.

**Keywords:** cohort studies, infant, micronutrients, nutrition, vitamin D

## Abstract

**Objective:**

The objective of this study was to examine risk factors for vitamin D deficiency and determine the association of vitamin D status with child growth and incidence of common morbidities among Tanzanian infants.

**Methods:**

A prospective cohort of 581 Tanzanian infants born to human immunodeficiency virus (HIV)-uninfected mothers had serum 25-hydroxyvitamin D assessed at 6 weeks and 6 months of age. Infants were seen at monthly clinic visits for growth monitoring until 18 months of age. Physicians examined infants every 3 months or when an illness was noted to document morbidities.

**Results:**

The prevalence of vitamin D deficiency (<20 ng/mL) declined from 76.4% at 6 weeks of age to 21.2% at 6 months. Infants who were exclusively breastfed at 6 weeks of age had 2.05 (95% confidence interval 1.11–3.79; *P* = 0.02) times the risk of vitamin D deficiency as compared formula-fed infants. After multivariate adjustment, there was no association of vitamin D status at 6 weeks or 6 months with the incidence of stunting, wasting, or underweight. There was also no association of low vitamin D with the incidence of diarrhea, upper respiratory infection, acute lower respiratory tract infection, or malaria.

**Conclusions:**

Vitamin D deficiency is common during early infancy, particularly among exclusively breastfed infants; however, these observational data suggest it may not be an important contributor to morbidity and growth among the general population of Tanzanian infants. Future studies of vitamin D among high-risk infants, including those with low birthweight and exposed to HIV, may be warranted.

**What Is Known**

- Infants are at high risk for vitamin D deficiency due to limited sun exposure and low dietary intake via human breastmilk.
- Dark skin pigmentation is a risk factor for vitamin D deficiency.
- Severe vitamin D deficiency can cause nutritional rickets.
- A previous cohort study conducted in Tanzania determined human immunodeficiency virus (HIV)-exposed uninfected infants with severe vitamin D deficiency at 6 weeks of age had increased risk of wasting.

**What Is New**

- Vitamin D deficiency is common, particularly during the early weeks of life among HIV-unexposed Tanzanian infants.
- Vitamin D status among Tanzanian infants is primarily determined by breastfeeding status and season.
- Poor vitamin D status does not appear to be an important contributor to morbidity and growth among HIV-unexposed Tanzanian infants.

**See Commentary to Article Entitled “Vitamin D Deficiency Is Not Associated With Growth or the Incidence of Common Morbidities Among Tanzanian Infants” by Mager on page 357.**

Globally, it is estimated that 1 billion people are vitamin D deficient and nearly 50% of the global population is affected by vitamin D insufficiency (1,2). For most individuals, 50% to 90% of the body's vitamin D is produced by sun exposure of the skin while the remaining proportion is obtained from the diet (2). As a result, the risk of vitamin D deficiency is highly influenced by sun exposure, latitude, skin-covering clothing, and skin pigmentation (2,3). Infants are known to be at particularly high risk for vitamin D deficiency due to low rates of sun exposure and low dietary intake via human breastmilk, particularly if the mother is vitamin D deficient (4,5).

The link between severe vitamin D deficiency during childhood with nutritional rickets (which can cause significant growth faltering) has long been recognized; however, the effect of less severe forms of deficiency on linear growth and weight gain during infancy is not well established (1). To date, research on infant growth outcomes has primarily focused on the role of maternal vitamin D status in pregnancy, whereas less research has focused on infant vitamin D status (6,7). In particular, research is needed in low- and middle-income countries (LMICs) where both linear growth faltering during infancy and vitamin D deficiency are common (8). We previously published the first cohort study of infant vitamin D status and anthropometric growth in sub-Saharan Africa and found that among Tanzanian infants born to human immunodeficiency virus (HIV)-infected mothers, 58.6% of infants were vitamin D deficient (25-hydroxyvitamin D (25(OH)D) levels <20 ng/mL) at 6 weeks of age (9). HIV-exposed uninfected infants with severe vitamin D deficiency (25(OH)D) levels <10 ng/mL) at 6 weeks of age had significantly increased risk of incident wasting, but there was no association with other anthropometric outcomes. Nevertheless, it is unclear whether these growth findings among HIV-exposed infants are generalizable to the substantially larger population of infants born to HIV-uninfected mothers in sub-Saharan Africa.

We therefore performed a prospective cohort study of Tanzanian HIV-unexposed infants to evaluate the relationship of vitamin D status at 6 weeks and 6 months of age with growth and common childhood morbidities. Our results are intended to inform whether infant vitamin D supplementation trials for growth improvement should be pursued among the general infant population in Tanzania and similar settings in sub-Saharan Africa.

## METHODS

### Parent Trial Design

The parent trial for this prospective cohort study was a randomized, double-blind, factorial-designed, placebo-controlled trial of infant zinc and multivitamin supplementation conducted in Dar es Salaam, Tanzania from August 2007 to May 2011 (clinicaltrials.gov NCT 00421668) (10). Briefly, infants born to HIV-uninfected mothers were randomly assigned to one of four study arms (factorial design of zinc, multivitamins, and placebo) between 5 and 7 weeks of age. The multivitamin contained vitamin C, vitamin E, thiamine, riboflavin, niacin, vitamin B6, folate, and vitamin B12, and zinc capsules contained 5 mg of zinc. Infants were excluded from the trial if they were of multiple gestation or had a serious congenital anomaly.

### Study Population and Serum 25-Hydroxyvitamin D Quantification

Serum samples included in this vitamin D study were selected from infants enrolled in a substudy of biomarkers of environmental enteric dysfunction (n = 590) (11). Infants were eligible for the environmental enteric dysfunction and subsequently the vitamin D study were randomly sampled if they had a blood sample available at 6 weeks and 6 months of age and were not stunted (length-for-age *z* score [LAZ] ≥ −2) at the baseline 6-week visit. A total of 581 children met these criteria and had serum samples with adequate volume available for the vitamin D substudy. We used high-performance liquid chromatography tandem mass spectrometry (HPLC-MS/MS) at Boston Children's Hospital using an API-5000 (AB Sciex, Foster City, CA) was used to quantify serum 25(OH)D concentration from 6-week and 6-month blood samples (12). Day-to-day coefficient of variation ranged from 5.6% to 8.5%.

### Data Collection

Mothers and infants were followed at monthly clinic visits for 18 months after randomization. At the baseline visit study nurses conducted a structured interview to collect information on demographic characteristics. A household asset score was calculated as the sum of asset items that included a sofa, television, radio, refrigerator, and fan (13). At all study visits nurses asked infant feeding practices in the previous week including breastfeeding status and frequency and introduction of other foods. Exclusive breastfeeding was defined as feeding a child with breast milk only without any additional foods.

Study physicians performed a clinical exam every 3 months and/or when acute complaints of illness were noted by nurses at monthly visits. Mothers were also encouraged to bring their child to the study clinic for sick visits when the child was unwell. Diarrhea was defined as ≥3 loose or watery stools within a 24-hour period. Acute upper respiratory infection was defined as pharyngitis or rhinitis (both without fast breathing or chest indrawing). Acute lower respiratory infection was defined as cough or difficulty breathing, rapid respiratory rate (based on the same definition described previously), and either a fever of >38.3°C or chest retractions. Clinical malaria was symptomatically diagnosed with/without laboratory testing for parasitemia following integrated management of childhood illnesses guidelines to minimize malaria-related deaths (14).

Nurses collected infant length and weight measurements using a digital infant balance (Tanita, Japan) and a rigid length board with a movable foot piece at all study visits. LAZ, weight-for-length *z* score (WLZ), and weight-for-age *z* score (WAZ) were calculated using World Health Organization child growth standards (15). Stunting, wasting, and underweight were defined as a LAZ, WLZ, and WAZ of 2 or more standard deviations (SDs) below the World Health Organization population median, respectively (15).

### Statistical Methods

Based on the distribution of 25(OH)D and commonly used clinical cutoffs, we categorized 6-week vitamin D into 3 categories <10 ng/mL,10–19.9 ng/mL, and ≥20 ng/mL (16). Similarly, we categorized 6-month vitamin D into 3 categories based on common clinical cutoffs <20 ng/mL, 20–30 ng/mL, and ≥30 ng/mL (16). We first determined the cross-sectional association of demographic and infant characteristics with severe vitamin D deficiency (<10 ng/mL) at 6 weeks of age and vitamin D deficiency (<20 ng/mL) at 6 months of age using log-binomial models to calculate relative risk estimates (17). Multivariate log-binomial models were fit including all variables with a *P* <0.20 in univariate analysis (18).

Anthropometric growth curves stratified by 25(OH)D category were constructed using mixed effects models using restricted cubic splines with knots at 10 weeks, 2, 6, 9, and 16 months intervals after randomization and automatic knot selection using *P* < 0.05 to determine a parsimonious model (19). The association of 25(OH)D categories at 6 weeks and 6 months and change in anthropometric *z* scores (LAZ, WLZ, WAZ) were analyzed with generalized estimating equations. Change in anthropometric *z* scores between consecutive visits was treated as a longitudinal continuous outcome and 25(OH)D, infant age, along with other baseline covariates as explanatory variables. The potential nonlinear relationship of change in anthropometric *z* scores between consecutive visits over time was examined nonparametrically with restricted cubic splines for infant age (19). If a nonlinear relationship was found, we added the selected cubic spline terms to the above-specified model as covariates. To assess whether 25(OH)D status was associated with change in anthropometric *z* scores between consecutive visits over time, we included the interactions of 25(OH)D categories with infant age and its splines. The robust score test was then used to determine whether overall anthropometric *z* score trajectory differed by 25(OH)D categories at 6 weeks and 6 months of age.

We also examined the association of 25(OH)D categories with stunting, wasting, and underweight using proportional hazard models. Infants with the outcome of interest at the time of 25(OH)D assessment were excluded from stunting, wasting, and underweight analyses. We examined the relationship of 25(OH)D with the incidence rate of physician diagnoses of morbidities using Poisson regression to take in account differences in timing of routine and sick visits (20).

Potential confounders were selected a priori based on their potential association with infant vitamin D status and adverse child health outcomes baseline socioeconomic status, maternal health and nutrition indicators, and child nutrition factors. Confounders included maternal age, maternal education, parity, wealth tertile, child sex, study regimen, breast-feeding status, low birthweight (<2500 g), prematurity (<37 weeks), and season of 25(OH)D assessment. Missing data for covariates was retained in the analysis using the missing indicator method (21). All *P* values were 2-sided and *P* < 0.05 was considered statistically significant. Statistical analyses were performed using the SAS v 9.2 (SAS Institute Inc, Cary, NC).

### Ethics

Written informed consent was obtained from all mothers in the parent trial. Institutional approval was granted by the Harvard School of Public Health Human Subjects Committee (12875-02), the Muhimbili University of Health and Allied Sciences Committee of Research and Publications (2014-10-29/AEC/Vol.IX/28) and the Tanzanian National Institute of Medical Research (HQ/R.8a/Vol.IX/482).

## RESULTS

**FIGURE 1:**
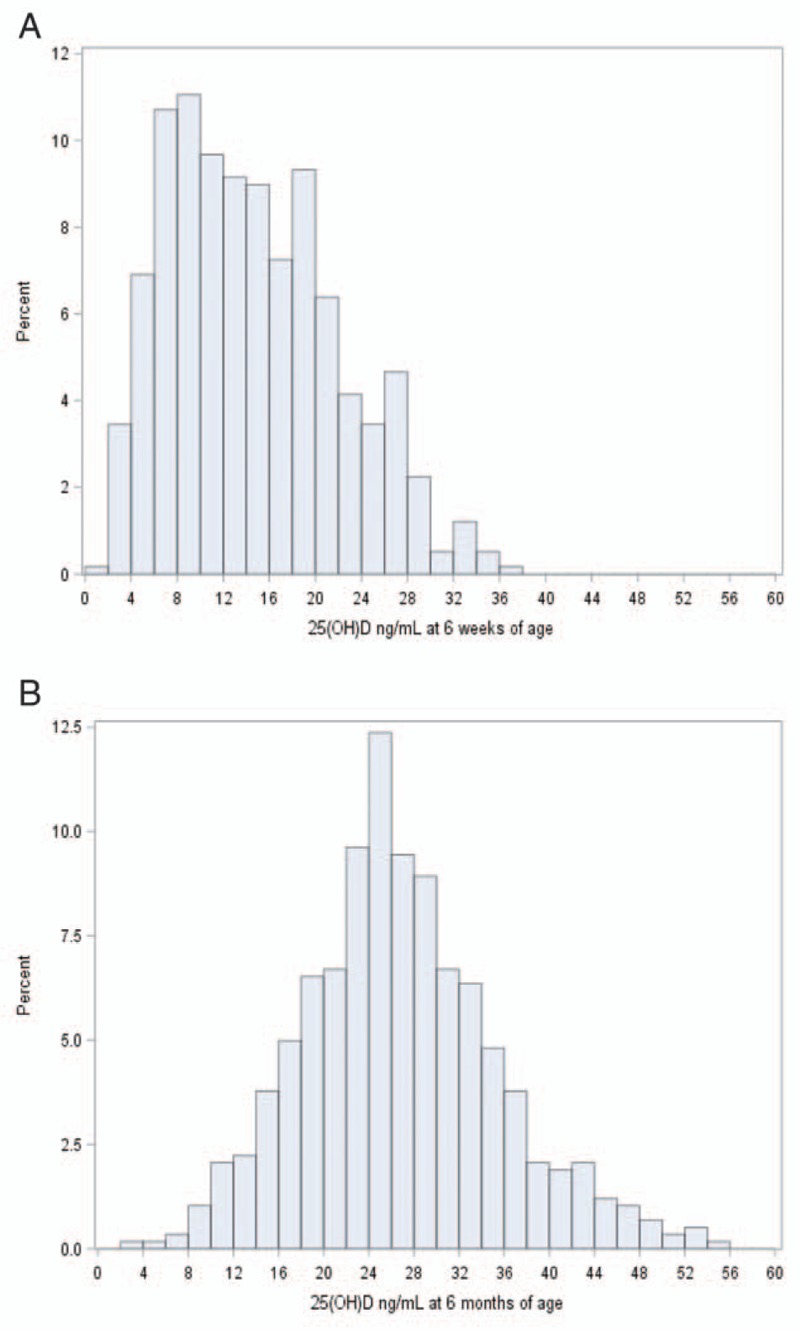
Distribution of 25(OH)D concentration (ng/mL) at 6 weeks (panel A) and 6 months (panel B) of age among 581 infants. 25(OH)D = 25-hydroxyvitamin D.

A total of 581 infants were enrolled in the vitamin D substudy. Maternal and child characteristics are presented in Table 1. Characteristics of infants included in the vitamin D study were similar to eligible infants (not stunted at 6 weeks) who did not have a vitamin D sample selected for analysis (n = 1661; Supplemental Digital Content, Table 1). The mean 25(OH)D concentration at 6 weeks of age was 14.5 ng/mL (SD 7.4) and 26.0 ng/mL (SD 8.7) at 6 months of age (Table 1). Figure 1A and B presents the distributions of 25(OH)D at 6 weeks and 6 months, respectively. The percentage of infants with vitamin D deficiency (<20 ng/mL) was 76.4% at 6 weeks and 21.2% at 6 months of age. The vast majority (92.1%) of infants had increased 25(OH)D levels from 6 weeks to 6 months of age with a mean increase of 12.2 ng/mL (SD 10.3).

### Risk Factors for Vitamin D Deficiency

Univariate and multivariate risk factor analyses for vitamin D deficiency (<20 ng/mL) at 6 weeks (Supplemental Digital Content, Table 2) and 6 months of age (Supplemental Digital Content, Table 3) are presented in the Supplemental Digital Content. In multivariate models, infants who were exclusively breastfed at 6 weeks had 2.05 (95% confidence interval [CI] 0.1.11–0.3.79; *P* = 0.02) times the risk of 25(OH)D levels <20 ng/mL as compared to formula-fed infants. Infants who were not exclusively breastfed and did not receive formula also appeared to be at increased risk for deficiency as compared to formula-fed infants (relative risk 1.78; 95% CI 0.96–3.30; *P* = 0.07). At 6 months of age, the postharvest season remained a significant risk factor for vitamin D deficiency (*P* < 0.01). Maternal age, maternal education, household wealth, child sex, birth order, low birth weight (<2500 g), and prematurity (<37 weeks) were not significantly associated with vitamin D deficiency at 6 weeks or 6 months of age.

### Vitamin D and Anthropometric Growth

**FIGURE 2:**
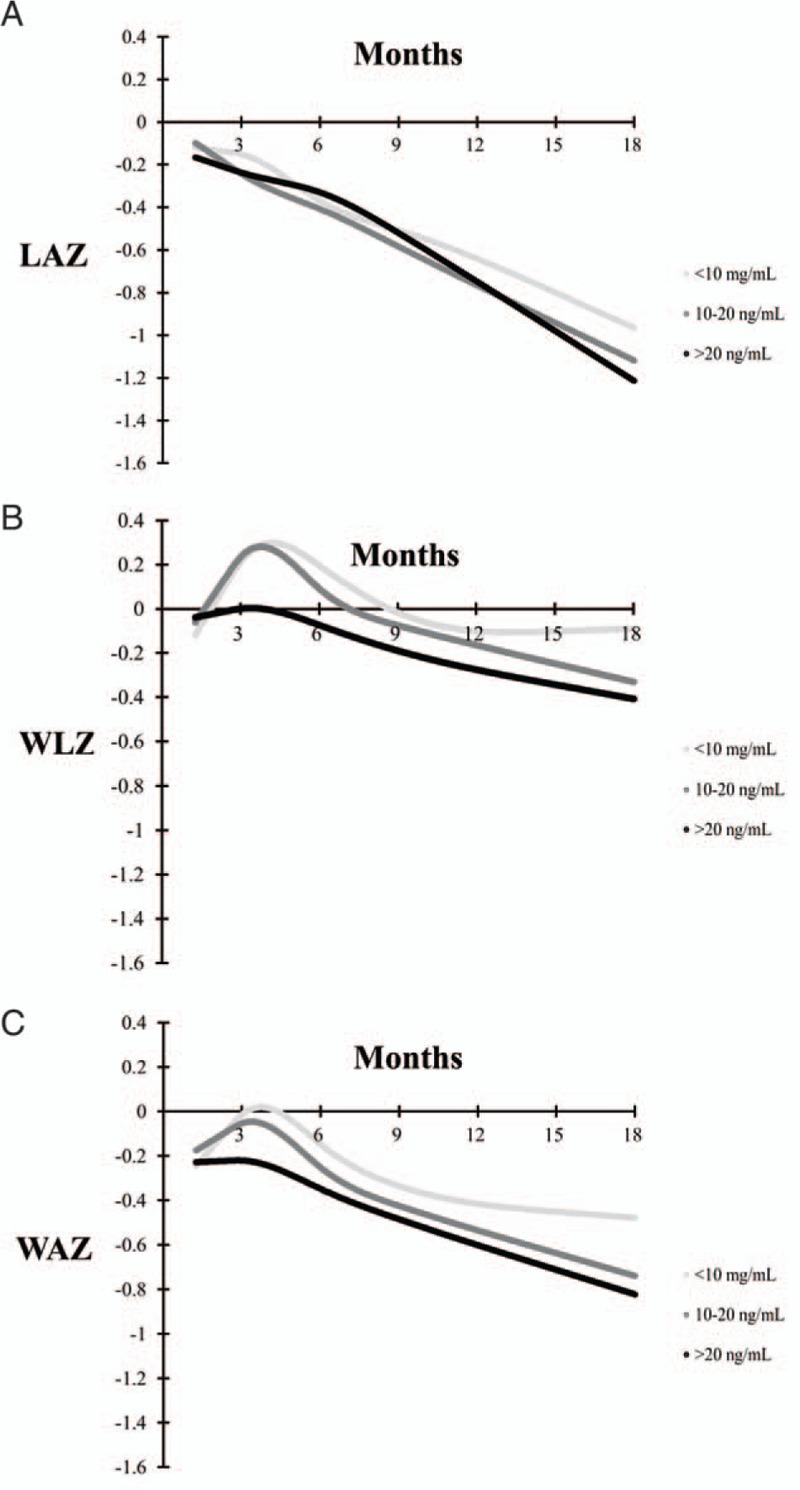
Mean length-for-age z score (LAZ) (panel A), weight-for-length z score (WLZ) (panel B), and weight-for-age z score (WAZ) (panel C) growth curves stratified by 6-week 25(OH)D concentration. There was no significant difference in trajectory of LAZ or WAZ by 6-week vitamin D status categories (*P* values for difference in trajectory >0.05). There was a significant difference in WLZ trajectory for infants with 25(OH)D concentrations at 6 weeks of >20 ng/mL as compared with infants with concentrations 10–20 ng/mL (*P* value for difference in WLZ trajectory <0.01). 25(OH)D = 25-hydroxyvitamin D.

Figure 2 presents LAZ, WLZ, and WAZ growth curves from 6 weeks to 18 months of age stratified by vitamin D status at 6 weeks of age. In multivariate analysis, the trajectory of WLZ significantly differed for infants with 25(OH)D concentrations of >20 ng/mL compared with those with concentrations 10 to 20 ng/mL (*P* value for difference in trajectory <0.01). There was a smaller increase in WLZ during the period of 6 weeks to 4 months for infants with vitamin D >20 ng/mL as compared to other groups, but all groups had similar WLZ at the end of the study period at 18 months of age. There were no differences in LAZ and WAZ trajectories by vitamin D status at 6 weeks of age (*P* values >0.05). There was also no association of vitamin D status at 6 weeks of age with incident stunting, underweight or wasting during follow-up (Table 2).

**FIGURE 3:**
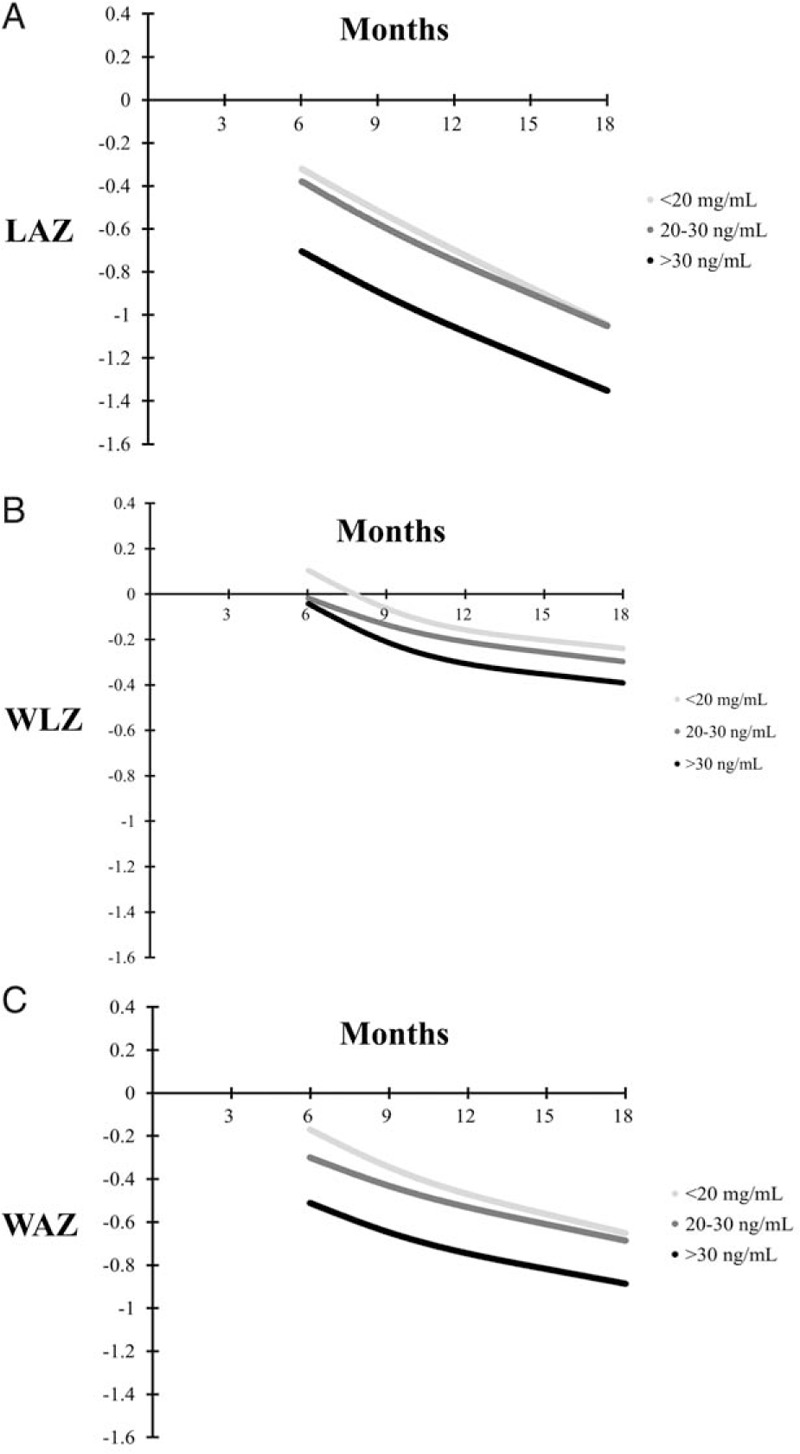
Mean length-for-age z score (LAZ) (panel A), weight-for-length z score (WLZ) (panel B), and weight-for-age z score (WAZ) (panel C) growth curves stratified by 6 month 25(OH)D concentration. There was no significant difference in trajectory of LAZ, WLZ, or WAZ by 6-month vitamin D status categories (*P* values for difference in trajectory >0.05). 25(OH)D = 25-hydroxyvitamin D.

Figure 3 presents LAZ, WLZ, and WAZ growth curves for 6 months to 18 months of age stratified by vitamin D status at 6 months of age. There were no differences in LAZ, WLZ, or WAZ trajectories by vitamin D status at 6 months of age in univariate and multivariate analyses (*P* values >0.05). There was also no association of vitamin D status at 6 months of age with incident stunting, underweight, or wasting (Table 2).

### Vitamin D and Morbidity

Table 3 presents the relationship of vitamin D status at 6 weeks and 6 months of age with the incidence of diarrhea, upper respiratory tract infection (URI), acute lower respiratory tract infection (ALRI), and clinical malaria. In multivariate analyses, there was no statistically significant association between vitamin D status at 6 weeks or 6 months of age with the incidence of diarrhea, URI, or clinical malaria (*P* > 0.05). In multivariate models, infants with 25(OH)D concentrations of 20 to 29.9 ng/mL at 6 months had decreased risk of ALRI as compared with infants with concentrations ≥30 ng/mL (incidence rate ratio 0.67; 95% CI 0.50–0.91).

## DISCUSSION

In this prospective cohort study of Tanzanian HIV-unexposed infant, we determined that vitamin D deficiency, defined by 25(OH)D levels <20 ng/mL, was present in >75% of infants at 6 weeks of age but declined to <25% by 6 months. Exclusively breastfed infants were at increased risk of vitamin D deficiency at 6 weeks of age and there was a consistent seasonal relationship at both 6 weeks and 6 months. There was no association of vitamin D status at 6 weeks or 6 months of age with LAZ and WLZ trajectory, nor with the incidence of stunting, wasting, or underweight. We also found no relationship of vitamin D status at 6 week or 6 months with incidence of diarrhea, URI, or clinical malaria, but infants with 25(OH)D concentrations ≥30 ng/mL at 6 months had increased risk of incident ALRI as compared with 20 to 29.9 ng/mL.

The high prevalence of vitamin D deficiency (76%) at 6 weeks of age among HIV-unexposed Tanzanian infants is consistent with our previous study in the same setting which found that 57% of HIV-infected and 60% of HIV-exposed uninfected Tanzanian infants were deficient at 6 weeks of age (9). Vitamin D deficiency is common during early infancy due to a combination of poor transplacental transfer of maternal vitamin D stores, low vitamin D content in human breastmilk, and limited sun exposure of young infants (22,23). Infants who were exclusively breastfed in our study had an increased prevalence of vitamin D deficiency at 6 weeks of age as compared to formula-fed infants and nonexclusively breastfed infants who did not receive formula. Infants who received formula at 6 weeks, which was likely vitamin D fortified, had the lowest risk of vitamin D deficiency. As for nonexclusively and non–formula-fed infants, the staple complementary food in Tanzania is maize-based porridge, which in itself is a poor source of vitamin D; however, cow's milk, condensed milk, meat, and other vitamin D containing foods are sometimes added or consumed separately (24). We did not have data available on sun exposure of infants.

Vitamin D is a potent immunomodulator with effects on both adaptive and innate immune responses and as a result may alter the incidence and severity of viral and bacterial infections (25). In this study, we found no statistically significant relationship of infant vitamin D status at 6 weeks or 6 months of age with incidence of diarrhea, URIs, or clinical malaria. In our previous study of Tanzanian HIV-exposed uninfected infants, which was also conducted in Dar es Salaam, we found no significant association of vitamin D at 6 weeks of age with incidence of diarrhea and ALRI, but 25(OH)D concentrations ≥30 ng/mL were associated with increased risk of clinical malaria (9). It is possible that our previous study findings indicating greater levels of vitamin D may increase risk of malaria were due to confounding, occurred by chance, or are specific to HIV-exposed populations. Only one randomized trial has examined the effect of infant vitamin D supplementation on the incidence and severity of morbidities in LMICs (26). In this trial conducted among Afghani infants (1–11 months of age) who were hospital admitted with pneumonia, vitamin D supplements significantly increased the incidence of repeat episodes of pneumonia as compared with placebo (26). In this study, we also found infants with 25(OH)D concentrations ≥30 ng/mL at 6 months of age had increased risk of ALRI as compared to infants with concentrations of 20 to 29.9 ng/mL. Consequently, the existing, even though very limited, observational and trial evidence do not support vitamin D supplementation among the general population of infants in LMICs to reduce common morbidities.

We also found no significant relationship of low vitamin D status at 6 weeks and 6 months with anthropometric growth, which is in contrast to our previous study where we found that HIV-exposed infants with 25(OH)D concentrations <10 ng/mL at 6 weeks of age had significantly poorer WLZ trajectory and increased risk of wasting during the first 2 years of life. Similarly in the current study, we found HIV-unexposed Tanzanian infants with 25(OH)D levels <20 ng/mL at 6 months had slightly increased risk of wasting but the results were not statistically significant. Of note, the incidence of wasting among HIV-unexposed infants in this study was less than half of that seen in our previous study of HIV-exposed uninfected infants and therefore the current study may have had inadequate power to detect a moderate difference in wasting. Limited data exist about the effect of vitamin D supplementation on weight gain and body composition. In 1 trial low birthweight (1.8–2.5 kg) Indian infants randomized to receive weekly vitamin D from birth had increased length, weight, and arm circumference at 6 months of age as compared to infants who received placebo (27). In addition, an observational study of Canadian infants found higher vitamin D concentrations during infancy to 3 years of age were associated with leaner body composition (28). Studies evaluating the association of child 25(OH)D status with body composition are warranted in LMICs given rapidly increasing rates of pediatric obesity and noncommunicable diseases in adulthood (29).

This study has a few limitations. First, the moderately small sample size may have led to inadequate power to detect modest effect sizes on growth and incidence of morbidities. Nevertheless, the direction of the point estimates and bounds of 95% confidence intervals suggest vitamin D supplementation is not likely to provide a large beneficial effect on growth or morbidity in the study population. Due to the observational nature of the study, we also cannot rule out residual confounding by SES, child feeding method, and other factors. Of note, we did not have data on maternal vitamin D status at 6 weeks or 6 months postpartum.

Vitamin D deficiency is highly prevalent among Tanzanian HIV-unexposed infants at 6 weeks of age but declines significantly by 6 months. We found no relationship of low blood concentrations of vitamin D at 6 weeks or 6 months of age with anthropometric growth or morbidity to 18 months of age. Due to the observational nature the study the findings cannot be considered causal, but they do not provide support vitamin D supplementation among the general population of Tanzanian infants to improve growth or to reduce risk for morbid conditions such as diarrhea, upper respiratory infections, and ALRIs. Future vitamin D supplementation studies and trials may be warranted among subsets of high-risk infants, including HIV-exposed uninfected and/or low birthweight infants, in Tanzania and similar LMIC settings (27,30).

## Supplementary Material

Supplemental Digital Content

## Supplementary Material

Supplemental Digital Content

## Supplementary Material

Supplemental Digital Content

## Figures and Tables

**TABLE 1:** Characteristics of 581 infants assessed for serum 25-hydroxyvitamin D concentration 6 weeks and 6 months of age

	Mean ± SD or frequency (%)
Maternal characteristics	
Maternal age, y	25.9 ± 4.9
Education	
None	10 (1.7)
Primary	428 (73.7)
Secondary or greater	140 (24.1)
Married/living with partner	517 (89.0)
Prior pregnancies	
None	195 (33.6)
1–3	372 (64.0)
≥4	11 (1.9)
Household asset score	
0–1	175 (30.1)
2–3	336 (57.8)
≥4	66 (11.4)
Child characteristics	
Male	279 (48.0)
Low birthweight (<2500 g)	16 (2.8)
Prematurity (<37 weeks gestation)	59 (11.0)
Exclusively breastfed at 6 weeks	390 (67.1)
Formula fed at 6 weeks	15 (2.6)
Exclusively breastfed at 6 months	10 (1.7)
No breastfeeding at 6 months	11 (1.9)
Formula fed at 6 months	14 (2.4)
Mean duration exclusive breastfeeding, mo	2.0 ± 1.7
Mean duration breast-feeding, mo	15.0 ± 4.6
LAZ at 6 weeks	−0.16 ± 0.99
WLZ at 6 weeks	−0.13 ± 1.21
WAZ at 6 weeks	−0.26 ± 0.88
Randomized regimen	
Placebo	149 (25.7)
Zinc	143 (24.6)
Multivitamins	146 (25.1)
Multivitamins + zinc	143 (24.6)
Season at 6 week 25(OH)D assessment	
Long rain (December–March)	187 (32.2)
Harvest (April–May)	94 (16.2)
Post-harvest (June–August)	201 (34.6)
Short rain (September–November)	99 (17.0)
Mean 25(OH)D concentration at 6 weeks, ng/mL	14.5 ± 7.4
Mean 25(OH)D concentration at 6 months, ng/mL	26.0 ± 8.7

LAZ = length-for-age *z* score; WAZ = weight-for-age *z* score; WLZ = weight-for-length *z* score; 25(OH)D = 25-hydroxyvitamin D.

**TABLE 2:** Prospective association of infant serum 25-hydroxyvitamin D at 6 weeks and 6 months of age with incident^∗^ stunting, wasting, and underweight

	No. with event/no. in category (%)	Unadjusted HR (95% CI)	*P*	Adjusted^†^ HR (95% CI)	*P*
25(OH)D at 6 weeks of age					
Stunting (LAZ < −2)					
<10 ng/mL	45/187 (24.1)	0.82 (0.53–1.26)	0.36	0.64 (0.41–1.01)	0.06
10–20 ng/mL	72/257 (28.0)	0.98 (0.67–1.45)	0.93	0.83 (0.55–1.23)	0.35
>20 ng/mL	39/137 (28.4)	Ref.		Ref.	
Wasting^*^ (WLZ < −2)					
<10 ng/mL	25/172 (14.5)	0.90 (0.51–1.62)	0.73	1.00 (0.54–1.89)	0.98
10–20 ng/mL	35/238 (14.7)	0.94 (0.55–1.62)	0.83	0.99 (0.57–1.74)	0.98
>20 ng/mL	21/130 (16.2)	Ref.		Ref.	
Underweight^*^ (WAZ < −2)					
<10 ng/mL	28/180 (15.6)	0.79 (0.46–1.36)	0.40	0.72 (0.40–1.28)	0.26
10–20 ng/mL	51/252 (20.2)	1.08 (0.67–1.75)	0.74	1.04 (0.63–1.70)	0.88
>20 ng/mL	25/134 (18.7)	Ref.		Ref.	
25(OH)D at 6 months of age					
Stunting (LAZ < −2)					
<20 ng/mL	23/111 (20.7)	0.83 (0.49–1.39)	0.48	0.78 (0.44–1.36)	0.38
20–30 ng/mL	42/239 (17.6)	0.75 (0.49–1.17)	0.21	0.72 (0.46–1.14)	0.17
>30 ng/mL	37/160 (23.1)	Ref.		Ref.	
Wasting^*^ (WLZ < −2)					
<20 ng/mL	15/108 (13.9)	1.23 (0.62–2.41)	0.63	1.27 (0.61–2.63)	0.53
20–30 ng/mL	27/242 (11.2)	1.05 (0.58–1.89)	0.87	1.03 (0.57–1.88)	0.92
>30 ng/mL	19/174 (10.9)	Ref.		Ref.	
Underweight^*^ (WAZ < −2)					
<20 ng/mL	16/111 (14.4)	0.84 (0.45–1.55)	0.57	0.90 (0.47–1.74)	0.76
20–30 ng/mL	35/252 (13.9)	0.82 (0.50–1.35)	0.43	0.80 (0.48–1.33)	0.39
>30 ng/mL	28/168 (16.7)	Ref.		Ref.	

HR = hazard ratio; LAZ = length-for-age z score; WLZ = weight-for-length z score; 25(OH)D = 25-hydroxyvitamin D.

^*^Analysis excludes infants with outcome of interest at the time of 25(OH)D assessment.

^†^Multivariate models adjusted for maternal age, maternal education, parity, wealth tertile, child sex, randomized regimen, low birthweight (<2500 g), prematurity (<37 weeks), breastfeeding method at 6 weeks of age, and season of 25(OH)D assessment.

**TABLE 3:** Association of 25-hydroxyvitamin D at 6 weeks and 6 months of age with incidence of diarrhea, upper respiratory tract infection, acute lower respiratory tract infection, and clinical malaria during follow-up

Vitamin D at 6 weeks of age	<10 ng/mL	*P*	10–19.9 ng/mL (Reference)	*P*	≥20 ng/mL
Diarrhea					
Mean diagnoses/year	1.12		1.01		1.30
Crude IRR (95% CI)	0.85 (0.67–1.08)	0.18	0.78 (0.63–0.97)	0.02	Ref.
Adjusted^*^ IRR (95% CI)	0.82 (0.63–1.07)	0.14	0.82 (0.65–1.03)	0.09	Ref.
URI					
Mean diagnoses/year	3.98		3.47		4.02
Crude IRR (95% CI)	0.97 (0.83–1.13)	0.68	0.86 (0.75–1.00)	0.05	Ref.
Adjusted^*^ IRR (95% CI)	0.94 (0.79–1.11)	0.46	0.87 (0.75–1.01)	0.08	Ref.
ALRI					
Mean diagnoses/year	0.74		0.73		0.85
Crude IRR (95% CI)	0.85 (0.64–1.14)	0.29	0.86 (0.65–1.14)	0.29	Ref.
Adjusted^*^ IRR (95% CI)	0.84 (0.61–1.17)	0.30	0.81 (0.60–1.08)	0.15	Ref.
Clinical malaria					
Mean diagnoses/year	0.98		0.87		0.97
Crude IRR (95% CI)	1.00 (0.76–1.31)	0.97	0.91 (0.70–1.18)	0.48	Ref.
Adjusted^*^ IRR (95% CI)	1.09 (0.80–1.49)	0.58	0.96 (0.72–1.26)	0.75	Ref.

Vitamin D at 6 months of age	<20 ng/mL	*P*	20–29.9 ng/mL (Reference)	*P*	≥30 ng/mL
Diarrhea					
Mean diagnoses/year	0.52		0.42		0.44
Crude IRR (95% CI)	1.21 (0.82–1.80)	0.34	1.01 (0.71–1.44)	0.96	Ref.
Adjusted^*^ IRR (95% CI)	1.20 (0.83–1.75)	0.33	1.03 (0.74–1.45)	0.85	Ref.
URI					
Mean diagnoses/year	2.92		2.76		2.97
Crude IRR (95% CI)	0.99 (0.82–1.19)	0.91	0.97 (0.84–1.12)	0.70	Ref.
Adjusted^*^ IRR (95% CI)	0.99 (0.83–1.19)	0.93	0.98 (0.85–1.14)	0.81	Ref.
ALRI					
Mean diagnoses/year	0.56		0.47		0.69
Crude IRR (95% CI)	0.82 (0.68–1.16)	0.27	0.72 (0.53–0.97)	0.03	Ref.
Adjusted^*^ IRR (95% CI)	0.81 (0.57–1.15)	0.23	0.67 (0.50–0.91)	0.01	Ref.
Clinical malaria					
Mean diagnoses/year	0.97		0.86		0.75
Crude IRR (95% CI)	1.31 (0.96–1.78)	0.08	1.20 (0.92–1.56)	0.17	Ref.
Adjusted^*^ IRR (95% CI)	1.33 (0.97–1.82)	0.08	1.22 (0.93–1.59)	0.15	Ref.

ALRI = acute lower respiratory tract infection; IRR = incidence rate ratio; URI = upper respiratory tract infection; 25(OH)D = 25-hydroxyvitamin D.

^*^Multivariate models adjusted for maternal age, maternal education, parity, wealth tertile, child sex, randomized regimen, breastfeeding method, low birthweight (<2500 g), prematurity (<37 weeks), and season of 25(OH)D assessment.
