# Supplementary material for: Vitamin D Deficiency Is Not Associated With Growth or the Incidence of Common Morbidities Among Tanzanian Infants
Source: J Pediatr Gastroenterol Nutr. 2017 Sep 22;65(4):467–74. doi: 10.1097/MPG.0000000000001658 (PMC5604126; doi:10.1097/MPG.0000000000001658)
Supplement: Supplemental Digital Content [file jpga-65-467-s001.docx]

Table 1. Characteristics of 581 infants assessed for serum 25(OH)D concentration 6 weeks and 6 months of age.

|  | Included in vitamin D study (n=581) | Eligible* for vitamin D study but did not have serum sample selected  (n=1,661) |
| --- | --- | --- |
|  | Mean ± SD or Frequency (%) | Mean ± SD or Frequency (%) |
| *Maternal Characteristics* |  |  |
| Maternal Age (years) | 25.9 ± 4.9 | 26.5 ± 4.9 |
| Education |  |  |
| None | 10 (1.7) | 24 (1.4) |
| Primary | 428 (73.7) | 1179 (71.0) |
| Secondary or greater | 140 (24.1) | 450 (27.1) |
| Married/living with partner | 517 (89.0) | 1494 (90.0) |
| Prior Pregnancies |  |  |
| None | 195 (33.6) | 494 (29.7) |
| 1-3 | 372 (64.0) | 1111 (66.9) |
| ≥ 4 | 11 (1.9) | 46 (2.8) |
| Household asset score |  |  |
| 0-1 | 175 (30.1) | 498 (30.0) |
| 2-3 | 336 (57.8) | 915 (55.1) |
| ≥ 4 | 66 (11.4) | 237 (14.3) |
| *Child Characteristics* |  |  |
| Male | 279 (48.0) | 838 (50.5) |
| Low birthweight (<2500g) | 16 (2.8) | 34 (2.1) |
| Prematurity (<37 weeks gestation) | 59 (11.0) | 187 (12.4) |
| Exclusively breastfed at 6 weeks | 390 (67.1) | 1111 (66.9) |
| Mean duration exclusive breastfeeding (months) | 2.0 ± 1.7 | 1.88 ± 1.5 |
| Mean duration breastfeeding (months) | 15.0 ± 4.6 |  |
| Length-for-age z-score at 6 weeks | -0.16 ± 0.99 | -0.10 ± 1.08 |
| Weight-for-length z-score at 6 weeks | -0.13 ± 1.21 | 0.08 ± 1.26 |
| Weight-for-age z-score at 6 weeks | -0.26 ± 0.88 | -0.07 ± 0.93 |
| Randomized regimen |  |  |
| Placebo | 149 (25.7) | 421 (25.4) |
| Zinc | 143 (24.6) | 418 (24.2) |
| Multivitamins | 146 (25.1) | 415 (25.0) |
| Multivitamins + zinc | 143 (24.6) | 406 (24.4) |

*LAZ ≥ -2 at 6 weeks of age

**Footnotes**

25(OH)D: 25-hydroxyvitamin D
