# Supplementary material for: Vitamin D Deficiency Is Not Associated With Growth or the Incidence of Common Morbidities Among Tanzanian Infants
Source: J Pediatr Gastroenterol Nutr. 2017 Sep 22;65(4):467–74. doi: 10.1097/MPG.0000000000001658 (PMC5604126; doi:10.1097/MPG.0000000000001658)
Supplement: Supplemental Digital Content [file jpga-65-467-s002.docx]

Supplemental Table. 2. Risk factors for 25(OH)D <20 ng/mL among Tanzanian infants (n=581) at 6 weeks of age

| Characteristic | Unadjusted relative risk (95% CI) | p-value | Multivariate relative risk (95% CI) | p-value |
| --- | --- | --- | --- | --- |
| *Maternal Characteristics* |  |  |  |  |
| Maternal age |  |  |  |  |
| <25 years | 1.00 (0.90-1.12) | 0.94 |  |  |
| 25-30 years | 0.98 (0.87-1.10) | 0.75 |  |  |
| >30 years | Ref. |  |  |  |
| Maternal education |  |  |  |  |
| No education/primary | 0.93 (0.36-2.42) | 0.88 |  |  |
| Secondary | Ref. |  |  |  |
| Wealth tertile |  |  |  |  |
| Poorest tertile | 0.97 (0.84-1.13) | 0.40* |  |  |
| Middle tertile | 0.97 (0.84-1.11) |  |  |  |
| Richest tertile | Ref. |  |  |  |
| *Child Characteristics* |  |  |  |  |
| Sex |  |  |  |  |
| Male | 1.06 (0.97-1.16) | 0.18 | 1.03 (0.99-1.07) | 0.08 |
| Female | Ref. |  | Ref. |  |
| Birth Order |  |  |  |  |
| First born | 1.16 (0.91-1.47) | 0.24 |  |  |
| 2^nd^ born plus | Ref. |  |  |  |
| Birthweight in grams |  |  |  |  |
| <2500 | 0.98 (0.74-1.31) | 0.90 |  |  |
| ≥2500 | Ref. |  |  |  |
| Gestational age in weeks |  |  |  |  |
| Preterm <37 weeks | 0.93 (0.78-1.10) | 0.40 |  |  |
| Term ≥37 weeks | Ref. |  |  |  |
| Feeding method at 6 weeks |  |  |  |  |
| Exclusive breastfeeding | 1.99 (1.07-3.71) | 0.03 | 2.05 (1.11-3.79) | 0.02 |
| Non-exclusive breastfeeding and no formula | 1.80 (0.96-3.38) | 0.06 | 1.78 (0.96-3.30) | 0.07 |
| Formula fed | Ref. |  | Ref. |  |
| Wasting at 6 weeks (WLZ < -2) |  |  |  |  |
| Yes | 1.09 (0.93-1.26) | 0.28 |  |  |
| No | Ref. |  |  |  |
| Underweight at 6 weeks (WAZ < -2) |  |  |  |  |
| Yes | 1.01 (0.75-1.36) | 0.97 |  |  |
| No | Ref. |  |  |  |
| Season at 25(OH)D assessment |  |  |  |  |
| Long rain (Dec-Mar) | Ref. |  | Ref. |  |
| Harvest (Apr-May) | 1.29 (1.12-1.49) | <0.01 | 1.33 (1.16-1.52) | <0.01 |
| Post-harvest (Jun-Aug) | 1.36 (1.21-1.54) | <0.01 | 1.40 (1.25-1.56) | <0.01 |
| Short rain (Sept-Nov) | 1.10 (0.93-1.30) | 0.25 | 1.10 (0.94-1.29) | 0.24 |

**Footnotes**

25(OH)D: 25-hydroxyvitamin D

CF: Complementary foods

*p-value for trend
