# Supplementary material for: Vitamin D Deficiency Is Not Associated With Growth or the Incidence of Common Morbidities Among Tanzanian Infants
Source: J Pediatr Gastroenterol Nutr. 2017 Sep 22;65(4):467–74. doi: 10.1097/MPG.0000000000001658 (PMC5604126; doi:10.1097/MPG.0000000000001658)
Supplement: Supplemental Digital Content [file jpga-65-467-s003.docx]

Supplemental Table 3. Risk factors for 25(OH)D <20 ng/mL among Tanzanian infants at 6 months of age

| Characteristic | Unadjusted relative risk (95% CI) | p-value | Multivariate relative risk (95% CI) | p-value |
| --- | --- | --- | --- | --- |
| *Maternal Characteristics* |  |  |  |  |
| Maternal age |  |  |  |  |
| <25 years | 1.12 (0.77-1.64) | 0.56 |  |  |
| 25-30 years | 0.84 (0.59-1.27) | 0.45 |  |  |
| >30 years | Ref. |  |  |  |
| Maternal education |  |  |  |  |
| No education/primary | 1.17 (0.33-4.13) | 0.81 |  |  |
| Secondary | Ref. |  |  |  |
| Wealth tertile |  |  |  |  |
| Poorest tertile | 0.93 (0.55-1.57) | 0.87* |  |  |
| Middle tertile | 1.02 (0.62-1.67) |  |  |  |
| Richest tertile | Ref. |  |  |  |
| *Child Characteristics* |  |  |  |  |
| Sex |  |  |  |  |
| Male | 0.98 (0.72-1.34) | 0.89 |  |  |
| Female | Ref. |  |  |  |
| Birth order |  |  |  |  |
| First born | 1.17 (0.83-1.65) | 0.37 |  |  |
| 2^nd^ born plus | Ref. |  |  |  |
| Birthweight in grams |  |  |  |  |
| <2500 | 1.15 (0.57-6.34) | 0.42 |  |  |
| ≥2500 | Ref. |  |  |  |
| Gestational age in weeks |  |  |  |  |
| Preterm <37 weeks | 0.95 (0.57-1.57) | 0.83 |  |  |
| Term ≥37 weeks | Ref. |  |  |  |
| Feeding method at 6 months |  |  |  |  |
| Exclusive breastfeeding | 0.94 (0.27-3.28) | 0.92 |  |  |
| Formula fed | 1.01 (0.36-2.78) | 0.99 |  |  |
| Partial breastfeeding and no formula | Ref. |  |  |  |
| No breastfeeding and no formula | 0.85 (0.24-3.02) | 0.81 |  |  |
| Stunting (LAZ < -2) at 6 mos. |  |  |  |  |
| Yes | 0.26 (0.07-0.99) | 0.05 | 0.26 (0.07-0.98) | 0.05 |
| No | Ref. |  | Ref. |  |
| Wasting (WLZ < -2) at 6 mos. |  |  |  |  |
| Yes | 1.03 (0.52-2.05) | 0.93 |  |  |
| No | Ref. |  |  |  |
| Underweight (WAZ < -2) at 6 mos. |  |  |  |  |
| Yes | 0.94 (0.45-1.96) | 0.87 |  |  |
| No | Ref. |  |  |  |
| Season at 25(OH)D assessment |  |  |  |  |
| Long rain (Dec-Mar) | Ref. |  | Ref. |  |
| Harvest (Apr-May) | 1.49 (0.82-2.71) | 0.19 | 1.51 (0.83-2.74) | 0.17 |
| Post-harvest (Jun-Aug) | 3.55 (2.30-5.49) | <0.01 | 3.55 (2.30-5.49) | <0.01 |
| Short rain (Sept-Nov) | 1.62 (0.90-2.90) | 0.11 | 1.64 (0.92-2.94) | 0.10 |

**Footnotes**

25(OH)D: 25-hydroxyvitamin D

CF: Complementary foods

*p-value for trend
